# The relationship between psychological distress and bullying victimisation among school-going adolescents in Ghana: a cross-sectional study

**DOI:** 10.1186/s13104-019-4300-6

**Published:** 2019-05-10

**Authors:** Diane Korkor Arhin, Kwaku Oppong Asante, Nuworza Kugbey, Mabel Oti-Boadi

**Affiliations:** 1 Department of Psychology, School of Social Sciences, University of Ghana, P. O. Box LG 84, Legon, Accra, Ghana; 2 Institute for Psychosocial Research on Child and Adolescent Wellbeing, Accra, Ghana; 3 Discipline of Psychology, University of KwaZulu-Natal, Durban, South Africa; 4 Department of Family and Community Health, University of Health and Allied Sciences, Hohoe Campus, Volta Region, Ghana; 5 General Studies Department, Ghana Technology University College, Accra, Ghana

**Keywords:** School-going adolescents, Bullying, Psychological health, Health promotion, Ghana

## Abstract

**Objective:**

One of the pervasive problems in schools that has adverse implication on the wellbeing of students, is bullying victimisation. However, within sub-Saharan Africa, only few studies have examined how psychological distress influences bullying victimisation. The purpose of this study was to explore the association between bullying victimization and psychological distress among school going adolescents in Ghana.

**Results:**

One hundred and ninety-eight (198) male and female students completed a series of self-report questionnaires measuring psychological distress (anxiety, stress and depression) and bullying victimisation in a cross-sectional survey. Pearson product-moment correlation coefficient and standard multiple regression were used to analyse the data. Findings revealed that bullying victimisation was positively associated with all the domains of psychological distress: depression (*r* = 0.35, *p* < 0.001), anxiety (*r* = 0.30, *p* < 0.001) and stress (*r* = 0.35, *p* < 0.001). Further analysis found depression to be the only significant predictor of bullying victimisation (β = 0.20; *t* = 2.01; *p* < 0.05). Taking into consideration that bullying victimization is a major problem among adolescents in schools, anti-bullying programmes should be implemented as this may promote improved wellbeing of school-going adolescents in Ghana.

## Introduction

One of the pervasive problems in schools that has adverse implication on the wellbeing of students, is bullying. The psychological implications of bullying have been found to be more problematic with children found at the early adolescence stage [1]. This could be alarming since growth at this stage of adolescence is very key in building a healthy personality in adulthood. Within the literature, depression was found to be positively correlated to bullying victimization [2, 3] but negatively correlated to bullying perpetration [1, 4].

Though the concept of bullying has received quite a number of attention in the developed countries [3, 5–7], only few studies have explored bullying victimisation among school going adolescents within Africa. Anecdotal evidence however shows a significant increase in the prevalence of bullying among school children and adolescents in Africa with a prevalence rates of 21–61% in South Africa [8], 78% in Malawi [9] and 59% in Ghana [1].

Literature has shown that victims of bullying experience poorer social, emotional, and physical health outcomes including signs of depression, suicidal ideation and loneliness [1, 9]. It has also been shown that social isolation and loneliness are associated with bullying victimisation [8, 10]. Recent systematic reviews have established a strong causal associations between bullying victimization and mental health problems such as depression, anxiety, poor general health and suicidal ideation and behaviours [11]. It has also been established that bullying victimisation is independently associated with significantly higher levels of psychological distress and reduced levels of emotional wellbeing [12]. Due to the dearth of research on bullying victimisation in developing countries, there remains a considerable gap in research that needs to be filled to provide an understanding into the scope and nature of the psychological effects of bullying victimisation as the findings in western countries may not necessarily be applied to the Ghanaian context. It has also been suggested that context and culture influences different patterns of victimization experiences among adolescents and young adults [13]. With no anti-bullying policy in place within Ghanaian schools in addition to the high prevalence of bullying reported among adolescents [1], there is therefore the need for a contextually relevant study to explore the various psychological distress that may be associated with bullying victimisation. The purpose of this study is to examine the relationship between psychological distress and bullying victimisation among school-going adolescents in Ghana. The findings of this study could help in the developing proactive anti-bullying intervention programmes for schools.

## Main text

### Methods

#### Study sample

A total of 198 students from three (3) junior high schools from the Ledzekuku Krowor Municipal Assembly (LEKMA) in the Greater Accra Region of Ghana were conveniently sampled to participate in the study. Participants age ranges from 11 to 19 years with an average age of 13.7 years (*SD* = 1.44). Over half of the sample (53.5%) were females with the remaining being males. Approximately 39.3% (n = 78) of the students were in first year, 34.8% (n = 69) in second year and the remaining 25.8% (n = 51) third year students.

#### Study design and protocol

A cross-sectional analysis carried out over 3 months (March–May 2018) where students were approached, and the purpose of the study explained. Those who agreed to participate signed a consent form. They were handed a questionnaire to fill out and return to the researcher. It took approximately 20 min to complete the questionnaire and was collected on the same day. Participants did not receive any form of compensation for their participation.

#### Study instrument

Two instruments were used in this study. These were the Depression, Anxiety and Stress Scale-21 (DASS-21) [14] and the bullying victimisation scale. The DASS-21 is a well-structured questionnaire consisting of 3 subscales of 7 items each measuring depression, anxiety and stress. The depression subscale assessed the presence of self-blame, pessimism and loss of enjoyment. The anxiety subscale assessed state of persistent apprehension and worry, accompanied by physical symptoms of sympathetic activation. The stress subscale assessed the state of over-arousal, tenseness and the inability to relax. The internal consistencies of the three subscales were 0.71, 0.79 and 0.81 for depression, stress and anxiety respectively. Items on the DASS-21 are scored on a 3-point Likert scale ranging from 0 = does not apply to me at all to 3 = applies to me very much or most of the time. The reliability of the DASS-21 has been established within the Ghanaian context [15–17]. In this study the Cronbach alphas for depression, stress and anxiety were 0.77, 0.82 and 0.88 respectively. Since, the DASS-21 distinguishes between depression, anxiety and stress as distinct manifestations of psychological distress, the total of the subscales were used in this study.

Bullying victimisation was assessed using 6 items selected from previous research which assessed bullying victimization in the past 30 days [18, 19]. The response options are rated on a 5-point Likert-type scale ranging from 0 (*never*) to 4 (*several times a week*) with higher scores on the bullying victimization shows elevated levels of victimization. Some of the items on the bullying victimization are: “I am called names, made fun of, or teased in a hurtful way” and “I was forced or threatened to do things I did not want to”. In this study the Cronbach alphas for bullying victimization was 0.72.

#### Statistical analysis

The Statistical Package for Social Sciences (SPSS) version 24.00 was used to analyse the data. Pearson’s correlation analysis was used to test the relationships among the study variables. A standard multiple regression analysis was conducted to identify which of the psychological distress dimension best predicts bullying victimisation. In the regression analysis, only variables that had significant correlation coefficients with the criterion variable were entered into the regression models. All statistical tests were performed using two-tailed examination, and a *p* value of 0.05 or less was considered statistically significant.

### Results

#### Relationship between domains of psychological distress and bullying victimisation

Our results in Table 1 showed that bullying victimisation was positively associated with depression (*r *= 0.35, *p *< 0.001), anxiety (*r* = 0.30, *p* < 0.001) and stress (*r *= 0.35, *p* < 0.001). These results suggest that higher levels of stress, anxiety and depression are associated with a corresponding elevated level of bullying victimisation among school-going adolescents.

**Table 1:** Correlation between psychological distress and bullying victimisation

Variables	*Mean*	*SD*	1	2	3	4
1. Bullying victimisation	4.80	1.80	1	0.35***	0.30***	0.35***
2. Stress	5.99	2.02	–	1	0.69***	0.65***
3. Anxiety	6.06	2.39	–	–	1	0.62***
4. Depression	6.01	2.11	–	–	–	1

***p < 0.001

#### Predictors of bullying victimisation

Standard multiple regressions were conducted to determine which variables significantly predicted bullying victimisations among school-going adolescents. The results, as presented in Table 2, showed that the regression model was significant (*F *= 9.40, *p* < 0.001); and accounted for 37.7% of the variance in bullying victimisation (*R*^2^ = 0.337). Further analysis revealed depression to be the only significant predictor of bullying victimisation (β = 0.20; *t* = 2.01; *p* < 0.05). The psychological distress dimension of stress and anxiety did not significantly predict bullying victimisation.

**Table 2:** Multiple regression analysis of the predictors of bullying victimisation

Model	*B*	*SE B*	*β*	*t*	*p* values
Depression	0.18	0.09	0.20	2.01	0.046*
Stress	0.12	0.11	0.12	1.10	0.272
Anxiety	0.10	0.10	0.10	0.98	0.330
*R* ^2^		0.337			
*F*		9.386***			

*B* unstandardised beta, *SEM* the standard error of the unstandardised beta, *β* standardised beta

*p < 0.05; ***p < 0.001, two-tailed

## Discussion

Findings from the study showed significant positive correlation between domains of psychological distress (depression, anxiety and stress) and bullying victimatization among school going adolescents in Ghana which suggest that elevated levels of psychological distress may pose as significant risk factors for bullying victimization [3, 20, 21]. The findings are not surprising as mental health problems among school going-adolescents predispose them to various risky behaviours including unsafe sexual behaviours and substance use [22, 23]. The symptoms of psychological distress could inhibit students’ ability to resist bullying as perpetrators of bullying victimization may target their colleagues who are unable to fight back.

We further examined the individual contributions of the three psychological distress domains and found depression to be the only significant predictor of bullying victimization among the students. This means that adolescent students who experience elevated depression levels are at higher risks of being bullied compared to those with lower levels of depression. Since depression as a mental health problem is characterized by sadness, isolation, loss of interest in previously enjoyed activities, lethargy and suicidal behaviours, students with depressive symptoms may appear as weak and unable to resist acts of aggression from their peers. This therefore makes depressed students targets of bullying. This finding is consistent with previous works among students which found psychological distress, social isolation and loneliness to be significantly associated with bullying victimization [8–13].

The implication of these findings are that prevalence of mental health problems among school going adolescents could serve as a significant risk factor for bullying victimization and creates the need for regular screening of in-school adolescents for common mental health problems. There is also the need for mental health promotion activities among adolescents in schools to ensure prevention, identification and early treatment to reduce their adverse impacts on academic and other wellbeing outcomes. Violence prevention policies and measures should be put in place in our schools to deal with perpetrators of bullying.

### Conclusion

This study demonstrated significant positive relationships among domains of psychological distress and bullying victimization among school-going adolescents which suggest the need for conscious efforts in identifying students at risk for bullying victimization especially those experiencing some form of psychological distress. Depression was the main contributing factor to the experience of more bullying victimization and as such intervention strategies should be tailored to this direction. There is the need for further studies to examine the mechanisms underlying the link between psychological distress and bullying victimization among school-going adolescents.

## Limitations

Our study is limited by the relatively small sample size and the use of a cross-sectional design as no causal inferences could be drawn between the variables used in this study. Another limitation was the convenient sampling which makes generalization difficult. A more representative sample could increase the generalizability of the results. There was also the potential of responses to be affected by social desirability as the study relied on self-report measures. However, previous studies mainly focused on the effects of bullying victimization of the health and wellbeing of adolescents whereas this study identified risk factors which may serve as a basis for future research on the risk factors for bullying victimization.

## Data Availability

The datasets used and/or analysed during the current study are available from the lead and corresponding authors on reasonable request.

## Abbreviations

DASS: Depression, Anxiety and Stress Scale
LEKMA: Ledzekuku Krowor Municipal Assembly
SPSS: Statistical Package for Social Sciences
SEM: the standard error of the unstandardised beta
